# RETRACTED: Deeparani, M.; Kalamani, M. Gynecological Healthcare: Unveiling Pelvic Masses Classification Through Evolutionary Gravitational Neocognitron Neural Network Optimized with Nomadic People Optimizer. *Diagnostics* 2023, *13*, 3131

**DOI:** 10.3390/diagnostics16162617

**Published:** 2026-08-18

**Authors:** M. Deeparani, M. Kalamani

**Affiliations:** 1 Department of Biomedical Engineering, Hindusthan College of Engineering and Technology, Coimbatore 641032, India; deepusabari1984@gmail.com; 2 Department of Electronics and Communication Engineering, KPR Institute of Engineering and Technology, Coimbatore 641407, India

The journal retracts the article titled, “Gynecological Healthcare: Unveiling Pelvic Masses Classification through Evolutionary Gravitational Neocognitron Neural Network Optimized with Nomadic People Optimizer” [1], cited above.

Following publication, concerns were brought to the attention of the publisher regarding similarities between this publication [1], and the previously published paper [2], originating from a different authorship group.

In accordance with the journal’s standard procedures, the Editorial Office and Editorial Board conducted an investigation, that confirmed substantial similarities between this publication and the above-mentioned source, including the underlying data, the description and composition of the dataset, and the reported ethical approval. While the authors cooperated with the investigation, they were unable to provide adequate supporting documentation to establish the authenticity, provenance, and ownership of the data reported in this study. Consequently, the Editorial Board has lost confidence in the reliability of the findings and decided to retract this publication [1], as per MDPI’s retraction policy.

This retraction was approved by the Editor-in-Chief of the journal *Diagnostics*.

The authors have been informed of this retraction but did not provide a comment on this decision.
